# Anticancer effect of salidroside on A549 lung cancer cells through inhibition of oxidative stress and phospho-p38 expression

**DOI:** 10.3892/ol.2014.1863

**Published:** 2014-02-10

**Authors:** JUN WANG, JIAN-ZHE LI, AI-XIA LU, KE-FEN ZHANG, BAO-JIANG LI

**Affiliations:** 1 Department of Oncology, The Central Hospital of Taian, Taian, Shandong 271000, P.R. China; 2 Department of Medical Laboratory, Taishan Sanatorium, Taian, Shandong 271000, P.R. China; 3 Department of Breast Surgery, The Central Hospital of Taian, Taian, Shandong 271000, P.R. China

**Keywords:** salidroside, lung cancer, reactive oxygen species, apoptosis, invasion, epithelial-mesenchymal transition

## Abstract

Oxidative stress is important in carcinogenesis and metastasis. Salidroside, a phenylpropanoid glycoside isolated from *Rhodiola rosea* L., shows potent antioxidant properties. The aim of the present study was to investigate the roles of salidroside in cell proliferation, the cell cycle, apoptosis, invasion and epithelial-mesenchymal transition (EMT) in A549 cells. The human alveolar adenocarcinoma cell line, A549, was incubated with various concentrations of salidroside (0, 1, 5, 10 and 20 μg/ml) and cell proliferation was detected by 3-(4,5-dimethylthiazol-2-yl)-2,5-diphenyltetrazolium bromide assay. Propidium iodide (PI) staining was used to determine the cell cycle by flow cytometry. Cell apoptosis was detected by Annexin V-fluorescein isothiocyanate and PI double-staining, and tumor invasion was detected by Boyden chamber invasion assay. Western blot analysis was performed to detect the expression of EMT markers, Snail and phospho-p38. The results showed that salidroside significantly reduced the proliferation of A549 cells, inhibited cell cycle arrest in the G0/G1 phase and induced apoptosis. Salidroside inhibited transforming growth factor-β-induced tumor invasion and suppressed the protein expression of Snail. As an antioxidant, salidroside inhibited the intracellular reactive oxygen species (ROS) formation in a dose-dependent manner in A549 cells, and depletion of intracellular ROS by vitamin C suppressed apoptosis by salidroside treatment. Salidroside was also found to inhibit the expression of phospho-p38 in A549 cells. In conclusion, salidroside inhibits cell proliferation, the cell cycle and metastasis and induces apoptosis, which may be due to its interference in the intracellular ROS generation, thereby, downregulating the ROS-phospho-p38 signaling pathway.

## Introduction

Lung cancer is a malignant tumor originating from normal bronchial epithelial cells. Non-small cell lung cancer (NSCLC) comprises the majority of lung cancer cases, with high occurrence and a low five-year survival rate of ~15%. Accumulating evidence has been previously documented concerning the molecular mechanisms underlying lung cancer initiation and progression, highlighting new targets for therapy. Defects in programmed cell death or apoptosis are hallmark features of cancer and have been implicated in lung tumorigenesis and drug resistance (1). Thus, inhibition of apoptosis offers a novel strategy for cancer treatment.

Oxidative stress is a major apoptotic stimulus in cancer cells, which have particularly high energy metabolism due to their rapid growth and proliferation. Therefore, reactive oxygen species (ROS) are excessively generated from a mitochondria source and lead to lipid peroxidation, DNA damage and, consequently, apoptosis in cells (2,3). By contrast, inhibition of oxidative stress also shows anticancer effects. Antioxidants, such as polyphenols, exhibit a wide variety of biological functions, including apoptosis induction, growth arrest and inhibition of DNA synthesis (4,5). Therefore, targeting the oxidative stress pathways through induction or inhibition, the generation of ROS may enhance the proapoptotic machinery of cancer cells and offer a novel strategy for treatment.

*Rhodiola rosea* is a traditional Chinese medicine and has long been used as an adaptogen for enhancing the body’s resistance to fatigue, stimulating the nervous system and preventing high altitude sickness (6). Salidroside, a phenol glycoside compound extracted from *Rhodiola rosea*, is a potent antioxidant. Salidroside has been reported to exert antidiabetic, neuroprotective and hepatoprotective effects (7–9). It has been hypothesized that salidroside may alleviate mitochondrial-generated ROS and manipulate mitochondrial-related apoptosis in a variety of cells (10). Moreover, salidroside has been found to exert an antiproliferation effect on a number of various cancer cells (11,12), and induce cell-cycle arrest and apoptosis in breast cancer (13).

The aim of the current study was to investigate the effects of salidroside on cell proliferation, the cell cycle, apoptosis, invasion and epithelial-mesenchymal transition (EMT) in the NSCLC A549 cell line. In addition, intracellular ROS levels and phospho-p38 expression were detected, and their association with A549 cells treated with salidroside was explored.

## Materials and methods

### Materials

Salidroside (purity, >99%) was purchased from the National Institute of Pharmaceutical and Biological Products (Beijing, China). Recombinant human transforming growth factor-β (TGF-β) was purchased from R&D Systems (Minneapolis, MN, USA). Dulbecco’s modified Eagle’s medium (DMEM) and fetal bovine serum (FBS) were obtained from Invitrogen Life Technologies (Carlsbad, CA, USA). 3-(4,5-Dimethylthiazol-2-yl)-2,5-diphenyltetrazolium bromide (MTT), vitamin C and 2′,7′-dichlorodihydrofluorescein diacetate (DCFH-DA) were purchased from Sigma-Aldrich (Sigma, St. Louis, MO, USA). Anti-Snail, -phospho-p38 and -β-actin antibodies were purchased from Santa Cruz Biotechnology, Inc. (Santa Cruz, CA, USA).

### Cell culture

The human alveolar adenocarcinoma cell line, A549, was purchased from the Institute of Biochemistry and Cell Biology, Shanghai Institutes for Biological Sciences, Chinese Academy of Sciences (Shanghai, China). Cells were cultured in DMEM media and supplemented with 10% FBS, at 37°C in a humidified incubator with 5% CO_2_.

### Cell viability assay

Cell viability was determined by MTT assay. Briefly, A549 cells at the logarithmic growth phase were randomly seeded into 96-well culture plates at a density of 1×10^3^ cells/ml and were cultured with 100 μl DMEM media (supplemented with 10% FBS) in each well. Cell adhesion was achieved and the cells were incubated with various concentrations of salidroside (0, 1, 5, 10 and 20 μg/ml) for 12, 24, 48 and 72 h. For cell viability assay, 10 μl MTT solution (5 mg/ml) was added to each well and incubated at 37°C for 4 h. Following centrifugation at 3,000 rpm for 10 min, the supernatant was removed to obtain the formazan pellet. Next, the pellet was dissolved completely with 100 μl DMSO. An ELISA plate reader (Ricso RK201, Shenzhen Ricso Technology Co., Ltd, Shenzhen, China)was applied to measure the absorbance at a wavelength of 570 nm, to determine the amount of pellet.

### Cell cycle analysis

A549 cells at the logarithmic growth phase were randomly seeded in 60-mm culture dishes. After reaching 50% confluence, cells were cultured in serum-free medium for 24 h to induce cell quiescence. Subsequently, cells were incubated with various concentrations of salidroside (0, 1, 5, 10 and 20 μg/ml) in complete medium. After 24 h, the cells were harvested by trypsinization followed by centrifugation at 2,000 rpm for 5 min. Next, cold 70% ethanol was added to cells for resuspension. Finally, 1 ml propidium iodide (PI) stain solution (PI, 20 μg/ml and DNase free RNase A, 100 μg/ml) was added to samples, which were analyzed on a FACScan (Becton-Dickinson, Franklin Lakes, NJ, USA) within 30 min. Data were acquired from 10,000 cells and processed using Lysis II software (Becton-Dickinson).

### Cell apoptosis assay

A549 cells were incubated with various concentrations of salidroside (0, 1, 5, 10 and 20 μg/ml) for 24 h. Subsequently, ≥2×10^5^ cells were harvested from each group for apoptosis assay by Annexin V-fluorescein isothiocyanate (FITC) and PI double-staining. Following centrifugation at 2,000 rpm for 5 min, the pellet was resuspended in 100 μl 1X binding buffer with 2.5 μl Annexin V and 5 μl PI (final concentration, 10 μg/ml). After incubation for 15 min in the dark, samples were subjected to apoptosis assay by flow cytometry, followed by data analysis using Lysis software. In total, ≥10,000 events were analyzed for each sample.

### Cell migration assay

The Boyden chamber invasion assay was performed to determine the *in vitro* migration capability of A549 cells. This experiment was performed in 24-well tissue culture plates with Transwell filter membrane. The lower side of the filters were coated with type I collagen (0.5 mg/ml) and the lower part of the filter contained low-serum media. In the upper part of the Transwell plate, 5×10^4^ cells were resuspended in 100 μl DMEM media, plated and incubated with salidroside (10 μg/ml) and/or TGF-β (100 ng/ml). After 24 h, cells on the upper surface of the filter were removed and cells that had migrated to the lower part were considered invasive cells. These cells were stained with hematoxylin and eosin (Sigma-Aldrich) and counted under an inverted light microscope (IX70, Olympus, Tokyo, Japan; magnification, ×200) as the number of migrated cells (invasion index). Each sample was assayed in triplicate and repeated twice.

### Measurement of ROS generation

Intracellular ROS levels were determined by a fluorescence plate reader using DCFH-DA. The cells on 24-well plates were treated with various concentrations of salidroside (0, 1, 5, 10 and 20 μg/ml) for 1, 3 and 6 h, and then incubated with DCFH-DA at 37°C for 30 min. Following the removal of DCFH-DA, the cells were washed with phosphate buffered saline. The fluorescence plate reader (FACScan, Tecan Deutschland GmbH, Crailsheim, Germany) was used to detect DCFH-DA-loaded cells. In order to determine whether apoptois in A549 cells by Salidroside is dependent on oxidative stress, a prominent water-soluble antioxidant, vitamin C (100 μM), was pretreated to scavenge ROS.

### Western blot analysis

Proteins of A549 cells were isolated and their concentrations were determined by bicinchoninic acid protein concentration assay kit (Beijing Biosea Biotechnology Co. Ltd., Beijing, China). Proteins (50 μg) were separated on sodium dodecyl sulfate-polyacrylamide gel electrophoresis gels (polyacrylamide concentration, 100 g/l) and electrophoretically transferred to a polyvinylidene fluoride (PVDF) membrane. The PVDF membrane was blocked with 3% bovine serum albumin at 37°C for 1 h, and probed with the mouse monoclonal antibodies against human Snail (1:1,000) and phospho-p38 (1:1,000). The horseradish peroxidase-conjugated rabbit anti-mouse IgG was used as secondary antibody at 1:1,000 dilution for 2 h at room temperature. The density of the targeted bands was visualized using the enhanced chemiluminescence method (Pierce^®^ ECL Plus Western Blotting Substrate, Pierce Biotechnology, Inc., Rockford, IL, USA) where Salidroside induces G1 phase cell cycle arrest in A549 cells. β-actin was used as an internal control.

### Statistical analysis

All quantitative data are presented as the mean ± standard deviation. Statistical analysis was performed using commercially available software (SPSS, version 14.0; SPSS, Inc., Chicago, IL, USA). An unpaired, two-tailed Student’s t-test was performed to compare the means of two groups. P<0.05 was considered to indicate a statistically significant difference.

## Results

### Salidroside inhibits the proliferation of A549 cells

**Figure 1:**
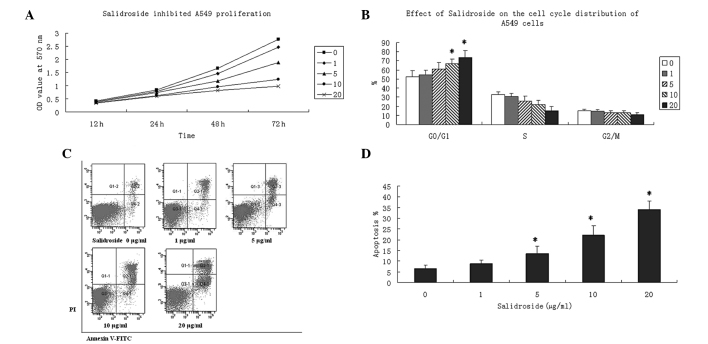
Effect of salidroside on the cell viability of A549 cells. (A) Cells were seeded onto 96-well culture plates and incubated with various concentrations of salidroside (0, 1, 5, 10 and 20 μg/ml) for 24 h. Cell proliferation was detected by MTT assay. Data are presented as the OD values at 570 nm wavelength and were obtained from at least three independent experiments. (B) Cells were seeded and incubated with various concentrations of salidroside (0, 1, 5, 10 and 20 μg/ml) for 24 h. PI (20 μg/ml) staining was performed to determine the percentages of cells in the G0/G1, S and G2/M phases. (C) Cell apoptosis was determined using Annexin V-FITC and PI double-staining. Salidroside treatment increased the apoptotic rate in A549 cells in a concentration-dependent manner. Images from three experiments are shown. (D) Apoptotic rates were analyzed in A549 cells treated with various concentrations of salidroside (0, 1, 5, 10 and 20 μg/ml) for 24 h. Annexin V^+^/PI^−^ and Annexin V^+^/PI^+^ populations were considered as apoptotic cells. Data are presented as the mean ± SD and were compared using a two-tailed, unpaired t-test. ^*^P<0.05, vs. the control group. PI, propidium iodide; FITC, fluorescein isothiocyanate; MTT, 3-(4,5-dimethylthiazol-2-yl)-2,5-diphenyltetrazolium bromide.

To evaluate the effect of salidroside on the cell viability of A549 cells, cells were simultaneously treated with various concentrations of salidroside (0, 1, 5, 10 and 20 μg/ml) for different time periods (12, 24, 48 and 72 h). A549 cells treated with DMEM media served as a normal control. The MTT assay revealed that salidroside treatment could inhibit A549 cell proliferation and decrease viable cells in a concentration- and time-dependent manner, which was demonstrated by lower OD values at 570 nm. Salidroside showed the most potent effect on cell viability at a 20-μg/ml concentration for all time points (Fig. 1A).

### Salidroside induces G0/G1 phase cell cycle arrest in A549 cells

To investigate the detailed mechanism of the underlying antiproliferative activity of salidroside, flow cytometry was used to determine cell cycle distribution. Serum starvation was performed on A549 cells to induce cell quiescence, followed by treatment with various concentrations of salidroside (0, 1, 5, 10 and 20 μg/ml) for 24 h. Salidroside significantly increased the percentage of cells in the G0/G1 phase at concentrations of 10 and 20 μg/ml (P<0.05). However, the percentage of cells in the S and G2/M phases remained unchanged following salidroside treatment (Fig. 1B). This assay indicated that NaHS inhibited the proliferation of A549 cells by inducing G0/G1 phase arrest.

### Salidroside increases apoptosis in A549 cells

To investigate whether decreased viability was caused by increased apoptosis by salidroside treatment, A549 cells were cultivated in the presence of salidroside (0, 1, 5, 10 and 20 μg/ml) for 24 h and double-stained with Annexin V-FITC and PI. Salidroside was found to increase the apoptotic rate of A549 cells in a concentration-dependent manner, and to significantly increase the apoptotic rate at concentrations of 10 and 20 μg/ml (Fig. 1C and D).

### Salidroside inhibits the invasion and expression of EMT marker protein, Snail

**Figure 2:**
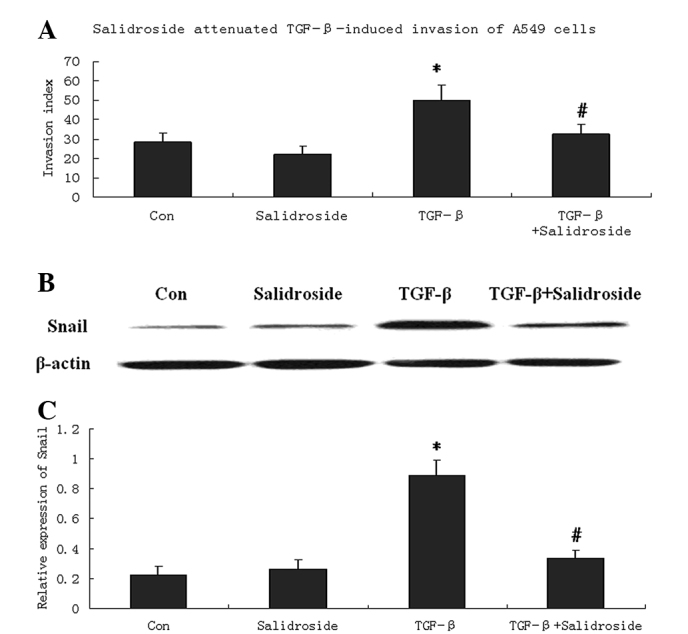
Salidroside inhibits the invasion and expression of the EMT marker protein, Snail, induced by TGF-β. (A) The proinvasive effect of TGF-β (100 ng/ml) was analyzed by Boyden chamber assay. A549 cells were incubated with salidroside (10 μg/ml) and/or TGF-β (100 ng/ml) for 24 h. Cells treated with Dulbecco’s modified Eagle’s medium served as control. Cell invasion was enhanced by TGF-β treatment, which was attenuated by salidroside. (B) Whole cell extracts of A549 cells were immunoblotted with the antibody against human Snail protein and β-actin served as loading control. One representative image is shown from three independent experiments. (C) Relative expression of Snail of the abovementioned four groups. The y-ordinate indicates the grayscale value of Snail normalized to that of β-actin. Data are presented as the mean ± SD and a two-tailed, unpaired t-test was performed. ^*^P<0.05, vs. the control group; ^#^P<0.05, vs. the TGF-β group. EMT, epithelial-mesenchymal transition; TGF-β, transforming growth factor-β.

To investigate whether salidroside inhibits the migration of tumor cells, the invasion capability of A549 cells was determined by Boyden chamber invasion assay. A549 cells were incubated with TGF-β to induce invasion. The results showed that TGF-β significantly increased the invasion index of A549 cells. Salidroside treatment significantly decreased the invasion index compared with cells treated with TGF-β (Fig. 2A). However, compared with the control cells, salidroside treatment alone only slightly decreased the invasion index, with no significant difference.

To investigate whether EMT is involved in the anti-invasive effect of salidroside, western blot analysis was performed to determine the expression of Snail, an EMT marker protein (14). In cells treated with TGF-β, Snail protein levels were significantly decreased by salidroside treatment. However, compared with control A549 cells, the levels of Snail protein remained unchanged following salidroside treatment (Fig. 2B).

### Salidroside decreases ROS generation in A549 cells

**Figure 3:**
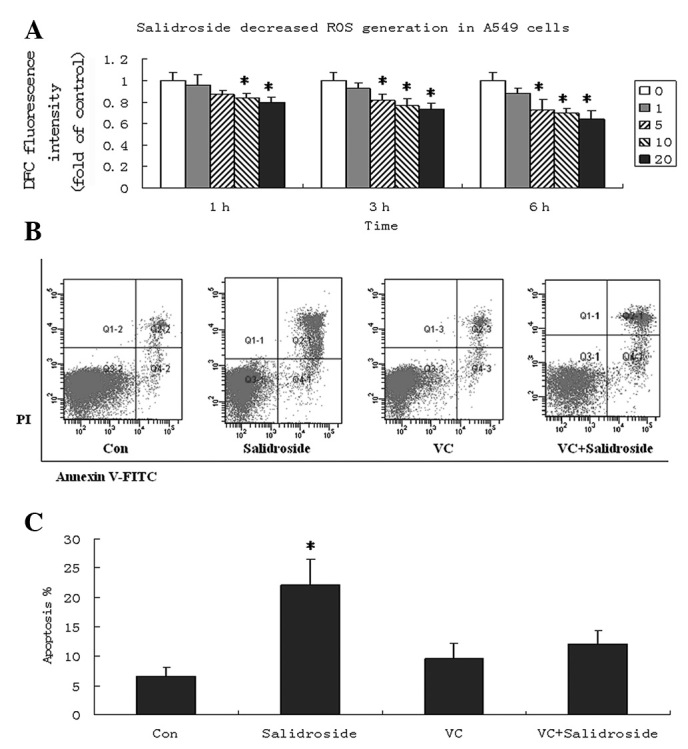
Salidroside decreases intracellular ROS in A549 cells. (A) Cells were treated with various concentrations of salidroside (0, 1, 5, 10 and 20 μg/ml) for 1, 3 and 6 h, followed by a 30-min incubation with 2′,7′-dichlorodihydrofluorescein diacetate at 37˚C for ROS detection. Data are presented as the fold increase compared with that of the control cells, and graphs present the mean ± SD. (B) VC pretreatment decreased the apoptosis of A540 cells induced by salidroside. VC (100 μM) was applied to A549 cells for 1 h. Subsequently, A549 cells were treated with salidroside (10 μg/ml) for 24 h and apoptosis was determined by Annexin V-FITC and PI double-staining. Images from three experiments are shown. (C) Apoptotic rates were analyzed in A549 cells. Annexin V^+^/PI^−^ and Annexin V^+^/PI^+^ populations were considered to be apoptotic cells. Data are presented as the mean ± SD and were compared using a two-tailed, unpaired t-test. ^*^P<0.05, vs. the control group. ROS, reactive oxygen species; VC, vitamin C; PI, propidium iodide; FITC, fluorescein isothiocyanate.

To investigate whether salidroside is involved in ROS generation and ROS-related apoptosis signaling in A549 cells, the fluorescence probe, DCFH-DA, was used to measure the intracellular ROS levels. The results showed that ROS levels were decreased by salidroside in a concentration- and time-dependent manner. Salidroside at 10 and 20 μg/ml significantly decreased the ROS levels in A549 cells after 1, 3 and 6 h (P<0.05; Fig. 3A).

The effect of intracellular ROS levels on apoptosis was further investigated following salidroside treatment. A549 cells were pretreated with 100 μM vitamin C (VC) for 1 h and cultured with salidroside (10 μg/ml). Pretreatment of A549 cells with VC significantly attenuated the apoptosis effect of salidroside and the apoptosis rate remained at ~10%, even at a 10-μM concentration (Fig. 3B and C). These results indicated that decreased intracellular ROS may be a mechanism underlying the cell death of A549 cells by salidroside.

### Salidroside decreases phospho-p38 MAPK expression

**Figure 4:**
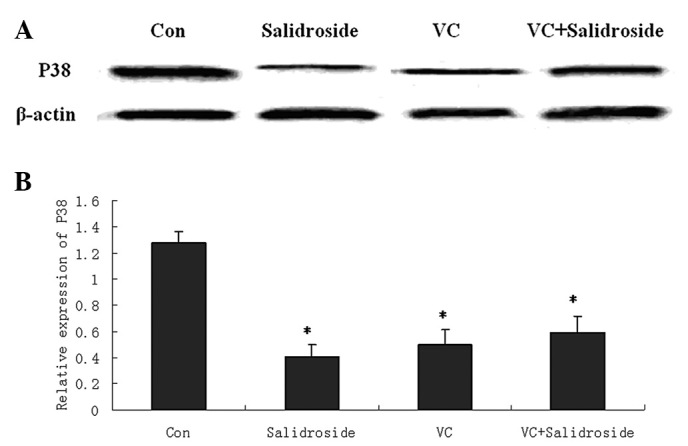
Salidroside decreases phospho-p38 protein expression in A549 cells. (A) Protein expression of phospho-p38 MAPK in A549 cells. A549 cells were pretreated with VC (100 μM) for 1 h and were then incubated with salidroside (10 μg/ml) for 24 h. Whole cell extracts were immunoblotted with the antibody against phospho-p38 and β-actin served as loading control. One representative image is shown from three independent experiments. (B) The density of each band was converted into a grayscale value and normalized to that of the internal control, β-actin. Data are presented as the mean ± SD. Phospho-p38 protein expression was significantly decreased in the salidroside group compared with the control group. VC pretreatment was also found to decrease phospho-p38 protein expression, which could not be further decreased by salidroside treatment. ^*^P<0.05, vs. the control group. VC, vitamin C.

To investigate the apoptosis signaling pathways underlying salidroside-treated A549 cells, phospho-p38 MAPK [one of the signaling proteins associated with oxidative stress (15)] was investigated for its protein expression. A549 cells were pretreated with 100 μM VC followed by salidroside treatment (10 μg/ml) for 24 h. Western blot analysis showed that salidroside significantly decreased phospho-p38 protein expression. VC pretreatment was found to also significantly decrease the phospho-p38 protein levels. However, salidroside could not further decrease phospho-p38 protein levels in VC-pretreated A549 cells (Fig. 4).

## Discussion

In the present study, salidroside, a phenol glycoside compound extracted from *Rhodiola rosea*, was found to show anticancer effects on *in vitro* cultured lung cancer A549 cells. These effects were demonstrated by suppressed cell proliferation, tumor invasion and EMT; arrested cell cycle; and reduced apoptosis. The underlying mechanisms may be associated with the inhibition of intracellular ROS generation and decreased phospho-p38 expression by salidroside. Salidroside decreased the intracellular ROS levels and phospho-p38 expression in A549 cells, which may be important for the anticancer activity observed in lung cancer cells.

The present study investigated the anticancer effects of salidroside on lung cancer cells, indicating a novel strategy for lung cancer treatment. Salidroside was found to reduce viable cells in a dose-dependent manner and the detailed mechanism lies in cell cycle arrest and induction of apoptosis. Following salidroside treatment, the percentage of cells in the G0/G1 phase was significantly increased. The results are consistent with those of a previous study demonstrating that salidroside caused G1- or G2-phase arrest in various cancer cell lines (11). Previously, salidroside has been found to demonstrate potent antiapoptotic effects in a variety of cells, including neurons (16), cardiomyocytes (17) and endothelia (18). However, a potent apoptotic effect of salidroside has been identified on lung cancer cells. Salidroside appears to exhibit antiapoptotic effects on non-tumor cells and apoptotic effects on tumor cells. For example, salidroside showed cytotoxic effects on breast cancer cells (13). Moreover, polyphenols, as antioxidants, also induce apoptosis in neutrophils (19), and liver (20) and breast (21) cancer cells. In this regard, salidroside inhibits survival signals, such as the Akt phosphorylation and mammalian target of the rapamycin pathway, and destructs mitochondrial integrity (20,21).

Tumor invasion is a multistage process that involves enhanced cell adhesion to extracellular matrix proteins. TGF-β acts as a tumor suppressor early in carcinogenesis, but in specific types of late stage cancer it is a prometastatic factor. TGF-β levels are elevated in cancer with more invasive phenotypes, and promote tumor invasion and metastasis (22). In the current study, TGF-β was incubated with A549 cells to induce invasion and significantly increase the invasion index of A549 cells. Salidroside was found to significantly decrease the invasion index of A549 cells induced by TGF-β. The observations are consistent with previous studies reporting that salidroside inhibits the migration and invasion of fibrosarcoma HT1080 cells, which was demonstrated by upregulated E-cadherin expression and downregulated β1-integrin expression (23). EMT is a vital step in the acquisition of epithelial cells with malignant phenotypes, including migration, invasion and metastasis to a new location (24). The results of the present study showed that following TGF-β treatment in A549 cells, salidroside significantly downregulated the expression of Snail, an EMT marker gene. This indicates that salidroside may suppress invasion through inhibition of the EMT process in A549 cells. It was also found that in control A549 cells without TGF-β, Snail protein levels remained unchanged following salidroside treatment. This may be explained by previous observations that salidroside suppresses TGF-β production and expression in high glucose-induced mesangial cell and experimental hepatic fibrosis rats, respectively (25,26).

The current study found that salidroside decreases ROS generation in A549 cells in a dose- and time-dependent manner. Pretreatment with antioxidant VC eliminates apoptosis induced by salidroside. This indicated that the capability of apoptosis induction by salidroside may rely on the high state of oxidative stress. Therefore, depletion of ROS by VC pretreatment reduced the sensitivity to salidroside. Salidroside was found to significantly decrease the protein expression of phospho-p38, a signaling protein associated with oxidative stress. However, in VC pretreated A549 cells, salidroside did not further decrease phospho-p38 protein levels. This indicated that high phospho-p38 expression is dependent on high levels of intracellular oxidative stress, which yields a high sensitivity of A549 cells to salidroside-induced apoptosis. Therefore, a decrease in phospho-p38 levels may be involved in apoptosis due to reduced ROS levels by salidroside. In a number of cell types, ROS-induced p38-MAPK activation is associated with increased apoptosis (27,28), which is contrary to the results of the current study. Salidroside is a phenol glycoside compound and shares a similar structure to polyphenols. As antioxidants, polyphenols have direct scavenging activity toward ROS and indirect antioxidant activity, the latter includes activation of antioxidant enzymes, such as glutathione peroxidase, glutathione S-transferase, catalase and NAD(P)H: quinone oxidoreductase-1 (4). Furthermore, the various fates of cells treated with polyphenols depend on their concentration, cell type, intracellular oxidative stress levels and stage of the pathological process (29). Therefore, further investigation is required to identify the detailed mechanism underlying the intercorrelation between ROS-induced p38-MAPK activation and apoptosis in lung cancer cells treated with salidroside, particularly the expression analysis of antioxidant enzymes.

In tumor cells, p38 MAPK is important in successful invasion and metastasis (30). Previously, p38siRNA has exerted an inhibitory effect on high glucose-induced EMT in tubular epithelial cells (31). In the present study, however, the correlation between the decreased protein expression of phospho-p38 and reduced tumor invasion by salidroside remains unknown and requires further study. The anticancer effects of salidroside must be further validated by *in vivo* animal studies.

In conclusion, salidroside shows anticancer effects in lung cancer cells. Decreased intracellular ROS and phospho-p38 may be the underlying mechanisms of salidroside activity. The present study indicates that salidroside is a promising therapeutic strategy for the treatment of lung cancer.
